# Transcriptional analysis of the response of *C. elegans* to ethanol exposure

**DOI:** 10.1038/s41598-021-90282-8

**Published:** 2021-05-26

**Authors:** Mark G. Sterken, Marijke H. van Wijk, Elizabeth C. Quamme, Joost A. G. Riksen, Lucinda Carnell, Laura D. Mathies, Andrew G. Davies, Jan E. Kammenga, Jill C. Bettinger

**Affiliations:** 1 Laboratory of Nematology, Wageningen University and Research, 6708 PB Wageningen, The Netherlands; 2 Department of Pharmacology and Toxicology, Virginia Commonwealth University, Box 980613, Richmond, VA 23298 USA; 3 Virginia Commonwealth University Alcohol Research Center, Richmond, VA USA; 4 Department of Biological Sciences, Central Washington University, Ellensburg, WA 98926 USA

**Keywords:** Gene regulation, Addiction, Addiction

## Abstract

Ethanol-induced transcriptional changes underlie important physiological responses to ethanol that are likely to contribute to the addictive properties of the drug. We examined the transcriptional responses of *Caenorhabditis elegans* across a timecourse of ethanol exposure, between 30 min and 8 h, to determine what genes and genetic pathways are regulated in response to ethanol in this model. We found that short exposures to ethanol (up to 2 h) induced expression of metabolic enzymes involved in metabolizing ethanol and retinol, while longer exposure (8 h) had much more profound effects on the transcriptome. Several genes that are known to be involved in the physiological response to ethanol, including direct ethanol targets, were regulated at 8 h of exposure. This longer exposure to ethanol also resulted in the regulation of genes involved in cilia function, which is consistent with an important role for the effects of ethanol on cilia in the deleterious effects of chronic ethanol consumption in humans. Finally, we found that food deprivation for an 8-h period induced gene expression changes that were somewhat ameliorated by the presence of ethanol, supporting previous observations that worms can use ethanol as a calorie source.

## Introduction

Alcohol is a commonly abused drug worldwide. Abuse of alcohol causes significant harm, contributing to approximately 5% of the global burden of disease^1^. Alcohol Use Disorder (AUD), the compulsive use of alcohol despite negative consequences, is the product of long-term misuse of alcohol (ethanol) that results in physiological changes in the brain and other organ systems. Such changes are likely to underlie both the pathological and the addictive characteristics of alcohol. Changes in gene expression can be important in the long-term consequences of ethanol exposure, so our goal is to understand the landscape of transcriptional responses to ethanol.

We use the nematode worm *C. elegans* as a model to study the physiological effects of ethanol. The molecular effects of ethanol in worms are directly relevant to the molecular effects of ethanol in mammals including humans^2^. *C. elegans* are intoxicated by exogenous ethanol, and they show a dynamic behavioral response to ethanol exposure. We observe behavioral depression within 10 min of exposure, which is a progressive slowing and loss of coordinated locomotion that reaches a plateau by 10 min of exposure^3,4^. The worms develop acute functional tolerance (AFT) to ethanol, which we first observe as an increase in locomotion speed between 10 and 30 min of exposure, despite a gradual increase in internal ethanol concentration over the exposure time^3,5,6^. We have extensively studied the mechanisms underlying these acute behavioral responses to ethanol^3,5–9^. However, we know less about the transcriptional modifications induced by ethanol, which are likely to underlie long term physiological adaptations to ethanol. We and others have observed that *C. elegans* develop physical dependence on ethanol over extended exposures; paradigms range between 6 and 24 h of exposure. In each case, dependence is observable as behavioral changes when the animals are withdrawn^5,10,11^. This suggests that there are important functional changes that occur over the exposure time, and we predict that modulation of transcription is likely to lead to a subset of these changes.

Here, we conduct a comprehensive analysis of the transcriptional responses across a time course of ethanol exposure in the nematode *C. elegans*. We examine the very acute responses at 30 and 60 min of exposure, responses after 2 h of exposure, and later responses after 8 h of continuous exposure. We observe waves of gene expression responses to ethanol, and we find that the largest number of gene expression changes are observable after 8 h of ethanol exposure. Among these are transcriptional changes due to food deprivation that is moderated by ethanol exposure, demonstrating that worms can use ethanol as a calorie source.

## Methods

### Nematode husbandry

*Caenorhabditis elegans* strain wild-type N2 (isolated in Bristol, UK) animals were maintained at 20 °C on nematode growth medium (NGM) plates in the presence of *Escherichia coli* strain OP50^12^.

### Age-synchronization

Populations were age-synchronized by allowing gravid adult hermaphrodites to lay eggs on a well-seeded plate for three hours. The adults were removed, and the resulting population was allowed to grow for three days at 20 °C until they were first-day adults.

### Drug treatment

Biological replicates, (*n* = 4) for a given timepoint, were generated on different days. 15 mL conical treatment tubes were prepared by adding M9+/− 100% ethanol to yield 0 mM or 400 mM ethanol. Approximately 2000 age-synchronized well-fed young adult worms were washed off of culture plates with M9 buffer into 15 mL conical tubes. The animals were allowed to settle for 2 min, and the supernatant was removed. Worms were washed with 5 mL M9, allowed to settle for 2 min, and the supernatant was removed. Each population of worms was divided into two halves and each half was added to a prepared treatment tube so that matched 0 mM and 400 mM treatments were generated. Worms were placed on a rotator at room temperature for the treatment time (0, 30, 60, 120, or 480 min). After the exposure was complete, tubes were removed from the rotator, the worms were allowed to settle for 2 min, and the supernatant was removed. Worms were washed twice with 5 mL M9, allowed to settle for 2 min, and the supernatant was removed. The resulting worm + M9 slurry was transferred to a microfuge tube and spun for one minute. Supernatant was removed to generate as dry a pellet as possible, and the worms were flash frozen in liquid nitrogen. Samples were then stored at − 80 °C. Worm samples were generated in the Bettinger laboratory (Richmond, Virginia, USA) and shipped frozen on dry ice to the Kammenga laboratory (Wageningen, NL) for processing and RNA analysis.

### RNA isolation

RNA was isolated as described previously^13^. Briefly, we used a Maxwell 16 AS2000 instrument with a Maxwell 16 LEV simplyRNA Tissue Kit (both Promega Corporation, Madison, WI, USA) following the manufacturer’s instructions with minor modifications. After the lysis step, 10 μL, instead of 25 μL, of a 20 mg/mL stock solution of proteinase K was added to each sample. Samples were incubated for 10 min at 65 °C and spun at 1000 rpm in a ThermoMixer (Eppendorf, Hamburg, Germany). Samples were cooled on ice for 1 min before loading into the cartridge, after which the standard protocol was followed. RNA samples were stored at − 80 °C until further use.

### cDNA synthesis, labelling, microarray hybridization and data extraction

The microarray preparation was conducted as described in the ‘Two-Color Microarray-Based Gene Expression Analysis; Low Input Quick Amp Labeling’-protocol, version 6.0 from Agilent (Agilent Technologies, Santa Clara, CA, USA). Based on RNA quantification by NanoDrop, we used 100 or 200 ng RNA as input for cDNA synthesis. The microarrays used were the Agilent *C. elegans* (V2) Gene Expression Microarray 4X44K slides. The microarrays were scanned with an Agilent High Resolution C Scanner using the recommended settings. After scanning, data was extracted with Agilent Feature Extraction Software (version 12.1.1.1), (https://www.agilent.com/en/product/mirna-microarray-platform/mirna-microarray-software/feature-extraction-software-228496).

### Normalization and data transformation

The microarray data was normalized using the Limma package in “R” (version 3.5.3, ×64)^14^. As recommended, the within array normalization used the ‘LOESS’ method and between-array normalization used the ‘quantile’ method^15,16^. After normalization, the normalized intensities were log_2_ transformed (I) and also a log_2_ ratio with the mean of each spot was calculated (R).

As we noticed a small dye-effect (from comparing the two dye-swaps) and batch effect based on an initial principal component analysis (< 5% of variance), we corrected the data for both dye and batch effects, by subtracting these effects as estimated based on the linear model

$${I}_{i,j}\sim {T}_{j}+{E}_{j}+{D}_{j}+{B}_{j}+{T}_{j}\times {E}_{j}+ {e}_{i,j}$$

where *I* is the log_2_ normalized intensity of spot *i* (1, 2, …, 45,220) of sample *j*, which was explained over timepoint *T* (0, 30, 60, 120, or 480 min of treatment), ethanol treatment *E* (0 mM or 400 mM), dye *D* (either Cy3 or Cy5), batch *B* (one out of four batches), and the interaction between timepoint and ethanol exposure, and residual variance *e*. Subsequently, the effects for dye and batch were subtracted from the normalized data.

### Data analysis and visualization

Data analysis was done in “R” (version 3.5.3, × 64) with custom written scripts^17^, accessible via https://git.wur.nl/published_papers/sterken_vanwijk_2020. The dplyr and tidyr packages were used for organization^18,19^, and plotting was done with ggplot2^20^. For gene-level analysis the annotations from WS258 were used, spots were re-mapped versus WS258 and probes with double or ambiguous (partial) hits were censored^21^. Transcriptome data were deposited at ArrayExpress under E-MTAB-9663.

### Quantitative PCR

As a confirmation of our microarray results, we performed quantitative PCR analysis on selected genes. We tested *mod-1* at 30 min of exposure*.* We tested *bas-1, che-3, fat-1, slo-1, unc-10, unc-13, unc-25, unc-47*, and *unc-49* at 480 min of exposure. We generated new cDNA from the biological samples tested in the microarray analysis. cDNA was made from 500 ng of RNA using the GoScript Reverse Transcriptase kit (Promega Corporation, Madison, WI, USA) following the recommended protocol with random hexamers (Thermo Scientific, Waltham, MA, USA). Gene expression was quantified by reverse transcriptase- quantitative PCR (RT-qPCR) using custom-designed primers (Supplemental Table 9). RT-qPCR was performed on the CFX Connect using iQ SYBR Green Supermix (Biorad, Hercules, CA, USA) according to the recommended protocol. Primer efficiencies were checked by testing dilution ranges in N2 populations. Gene expression in each sample was quantified for the gene of interest and two reference genes (Y37E3.8 & *rpl-6*) in technical duplicate^22^. Data was analyzed per time point. Data was transformed with the following formula:

$${Q}_{gene}={2}^{{Ct}_{control gene average}- C{t}_{gene}}$$

Relative gene expression was calculated using:

$$E=\frac{Qgene}{0.5*\left({Q}_{rpl-6}+{Q}_{Y37E3.8}\right)}$$

### Quantitative PCR statistics

To determine statistical significance over multiple samples we performed an ANOVA, as provided by R.

### Principal component analysis

To understand sources of variance we used principal component analysis, as provided in “R” by the *prcomp* function (scale. = TRUE). Principal component analysis was done on the log_2_ ratio with the mean transformed normalized data.

### Linear model

To determine which gene-expression was affected by the experimental variables, we used the following linear model

$${I}_{i,j}\sim {E}_{j}+ {e}_{i,j}$$

where *I* is the log_2_ normalized intensity of spot *i* (1, 2, …, 45,220) of sample *j*, which was explained over ethanol treatment *E* (0 mM or 400 mM) and residual variance *e*. This model was used per time-point separately (0, 30, 60, 120, and 480 min of 0 mM ethanol exposure). For each treatment time the combination of the four biological replicates was assayed.

To understand the between-timepoint dynamics, we calculated the difference between 0 and 400 mM ethanol exposed samples within the same batch and timepoint, and used the model

$${Y}_{i,k}\sim {T}_{k}+ {e}_{i,k}$$

where *Y* is the difference between 0 and 400 mM ethanol exposed samples of spot *i* (1, 2, …, 45,220) of batch *k*, which was explained over two subsequent timepoints *T* (0–30, 30–60, 60–120, or 120–480 min after the start of the experiment).

These linear models yielded information on gene expression changes within and between the five timepoints. The significances from both linear models were corrected for multiple testing using a Benjamini–Hochberg correction (as implemented in the *p.adjust* function in “R”)^23^. This was done on a general threshold of − log_10_(p) > 3.7, to standardize effect-size detection over the various factors. This threshold resulted in false-discovery rates between q = 0.0020 (0 mM ethanol at 480 min) and q = 0.123 (0–400 mM ethanol between 60–120 min). Combined, this set of transcriptional differences was composed of 5968 spots representing 3603 genes. Excluding the analyses involving the 480-min timepoint, the set includes 959 spots representing 660 genes (Supplemental Table 1; Supplemental Table 3; Fig. 3; Supplemental Table 4).

### Transcriptional time estimation

The relative transcriptional age between the samples was estimated by their transcriptional profiles. We used a temporal expression ruler generated by Snoek et al., 2014, that consisted of 2195 microarray spots (1266 genes) that were linearly up- or down-regulated (log_10_(p) > 6 and an absolute effect-size > 0.1 per hour) throughout development (41.5–72 h) (Supplemental Table 2)^24^. The data underlying the choice of the microarray spots can be obtained from E-MTAB-7019. In our studies, we only examined adults, so here we used these genes to estimate the general transcriptional progression in our animals, rather than developmental age. We compared the expression data of these genes in our data to the ruler to give transcriptional “age” per gene per sample using the function *predict* in “R” (version 3.5.3, ×64). We subsequently calculated the average age from the inferred ages per gene to obtain an estimated age for each sample. As these ages are strictly relative, the estimated ages were subsequently transformed into a normal standard distribution for relative age estimation and sample comparison.

### K-means clustering

To identify time-dependent patterns in the set of differentially expressed genes, we used k-means clustering. We used the log_2_ ratio with the mean transformed data (*R*) of the 959 spots that showed significant differences for the clustering analysis. This excluded the 480-min timepoints. To reliably identify patterns, we averaged values per spot per time per treatment over the four biological replicates (for the 0-, 30-, 60-, and 120-min timepoints). We used the “the elbow” method to determine the appropriate number of clusters by analyzing the within-cluster sum of square (WSS)^25^. The WSS measures the variability of the observations within each cluster. The number of clusters is generally considered appropriate when adding another cluster does not substantially improve the overall WSS. We used the function *fviz_nbclust* from the R package NbClust^26^. We clustered the selected spots using *kmeans* in R, using the Hartigan and Wong algorithm with 5 centers, 10,000 iterations and 50 random starts. These settings yielded a stable outcome when clustering was repeated (Supplemental Table 6).

### Enrichment analysis

We performed enrichment analyses for the linear model in R, using a hypergeometric test and various annotations. We used the following databases: the WS258 gene class annotations, the WS258 GO-annotation, anatomy terms, phenotypes, RNAi phenotypes, developmental stage expression, and disease related genes (www.wormbase.org)^27^; the ModERN transcription factor binding sites (http://epic.gs.washington.edu/modERN/)^28^, which were mapped to transcription start sites (as described by^29^) (Supplemental Table 5).

Enrichments were selected based on the number of genes in a category *n* > 3, number of overlapping genes in the query *n* > 2, and significance as determined by a Bonferroni-corrected hypergeometric test.

For the enrichment analysis for the k-means clusters we used g:profiler under the default setting but excluding electronic GO annotations^30^. We calculated all enrichments based on unique gene names (Supplemental Table 7).

### Transcription factor binding site enrichment analysis

To ask if there are transcription factor binding sites that are enriched in ethanol responsive genes, we used the ModERN database to obtain transcription factor binding sites in *C. elegans*^28^. We mapped transcription start sites according to Tepper et al*.*^29^. We performed enrichment analysis with a hypergeometric test on genes with the following criteria: FDR-corrected p value < 0.05, transcription factor binding site category > 3, and the size of the overlap > 2. After enrichment analysis and significance filtering, the enrichment fold change between different ethanol exposures was averaged.

## Results

### Transcriptional response to ethanol exposure

**Figure 1:**
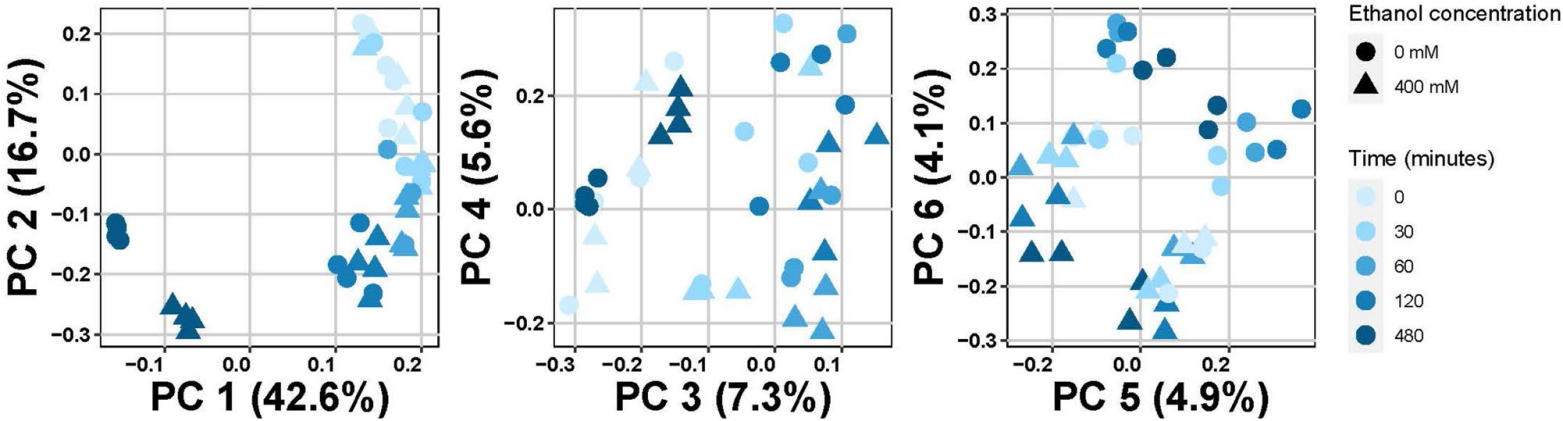
Principal component analysis. We identified six principal components contributing to the variation in the transcriptional responses in our assays (> 4% of variance). The colors indicate the time of 0 mM or 400 mM ethanol exposure (darker colors are longer exposure times). Circles: 0 mM ethanol exposed; Triangles: 400 mM ethanol exposed. The different principal components are depicted on the x- and y-axes.

To study the transcriptional response to ethanol exposure, we conducted a time course experiment. We exposed young adult, wild-type N2 worms for 0, 30, 60, 120, or 480 min to either 0 mM or 400 mM ethanol, isolated RNA, and analyzed the transcriptional response to ethanol using microarrays. We chose 400 mM ethanol for our exposure because we have extensively characterized the behavioral responses to ethanol at this concentration and have found that the genes affecting ethanol response behaviors at this concentration are also important in ethanol responses in mammals, including humans^2,9,31^. The microarray data was normalized and transformed for analysis. As a confirmation of the accuracy of our microarray results, we performed quantitative PCR of ten selected genes (Supplemental Fig. 1); the results confirmed our microarray analysis. Principal component analysis revealed that there was a large distinction (42.6% of the variance) between the group of 0–2 h exposure samples and the 8-h exposure samples. Within the 0–2 h exposure samples, the length of the experimental treatment accounted for another 16.7% of the variance in the samples, independent of the ethanol concentration. This indicated that the treatment paradigm itself, rather than ethanol exposure, caused most of the transcriptional effects (Fig. 1). 9% of the variance could be explained by the ethanol treatment (principal components 5 and 6, Fig. 1).

**Figure 2:**
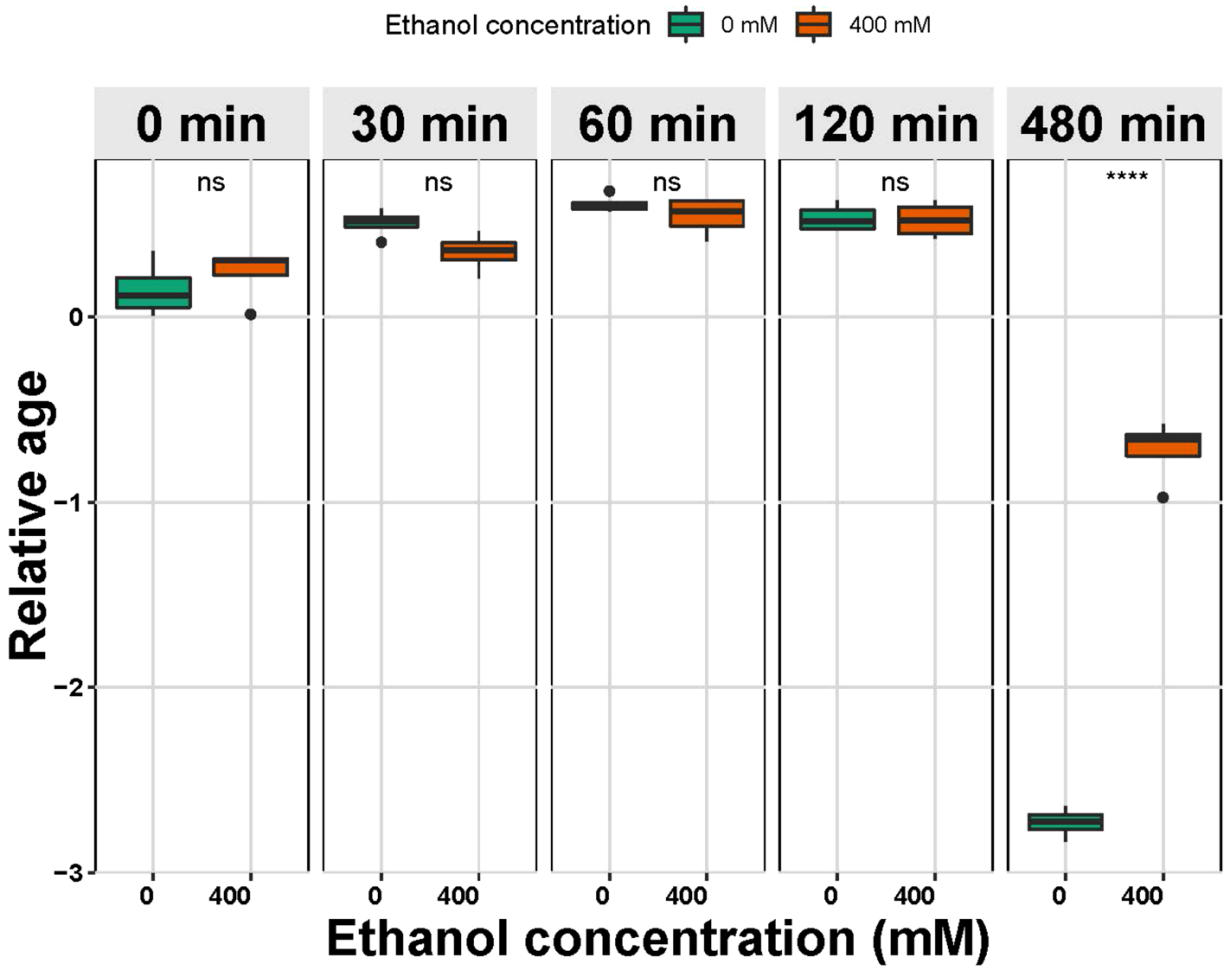
Effect of ethanol exposure on transcriptional time, presented as relative age. The relative age was computed by transforming the predicted ages (in hours) into the standard normal distribution. There was no significant difference in the “age” of animals due to ethanol exposure or to the treatment at 0, 30, 60, or 120 min of treatment. Animals undergoing the treatment for 480 min were significantly transcriptionally “younger” than animals treated for shorter times, although they were chronically 6–8 h older than the animals treated for shorter times. This suggests that this broad transcriptional delay is due to the food deprivation during the treatment. Ethanol exposure modified this transcriptional delay, suggesting that the animals are able to use ethanol as a calorie source, counteracting the starvation-induced effects on transcription. (one tailed t-test *p* < 0.0001).

We were interested in why our treatment had such strong effects on the transcriptomes of the animals. We used a method described by Snoek et al., 2014^24^, that used the behavior of genes whose expression vary linearly over time as a measure of the “developmental age” of animals to assess the response of animals to the duration of the treatment. Importantly, during the ethanol exposure, the animals were food-deprived, which is known to have profound effects on transcription^32^. Ethanol treatment has also been observed to variably delay embryonic and postembryonic development in worms^33^. We hypothesized that the 8-h exposure to ethanol and starvation might have global consequences on transcriptional activity, which we could detect by effects on the global “transcriptional age” of the animals. We generated a transcriptional “clock” by using data from Snoek et al., 2014^24^, selecting a subset of transcripts that are linearly up- or down-regulated across late stages of postembryonic development of worms (Supplemental Table 2). We used these transcripts to estimate the “transcriptional age” of the animals in our ethanol exposure experiment. We found that animals treated for 0, 30, 60, and 120 min were indistinguishable from each other in this analysis. However, when we looked at animals that had been treated for 8 h, we found that they displayed a different transcriptional “age” from those of the other treatments. The 8-h treatment animals were harvested when they were 6–8 h chronologically older than all the other animals in this study. Therefore, if there was no effect of treatment on transcriptional age, then we would expect to see that the 8 h treated animals would have a transcriptional profile that appeared in this analysis to be “older” than the profiles of the other samples. In contrast, we observed that both the 0 mM and 400 mM ethanol treated animals had transcriptional profiles that appeared to be “younger” than the other treatment groups (Fig. 2). Because both the groups were strongly affected, this suggests that the common condition of 8 h of food deprivation caused a delay in normal transcriptional aging. Intriguingly, we noted that the transcriptomes of the animals treated with 400 mM ethanol were significantly “older” than the transcriptomes of the 0 mM ethanol treated animals (Fig. 2), suggesting that the animals incubated with ethanol are able to use it to partially counteract the starvation-induced delay. This is evidence that they are using the ethanol as a calorie source, as has been previously reported^34–37^.

**Figure 3:**
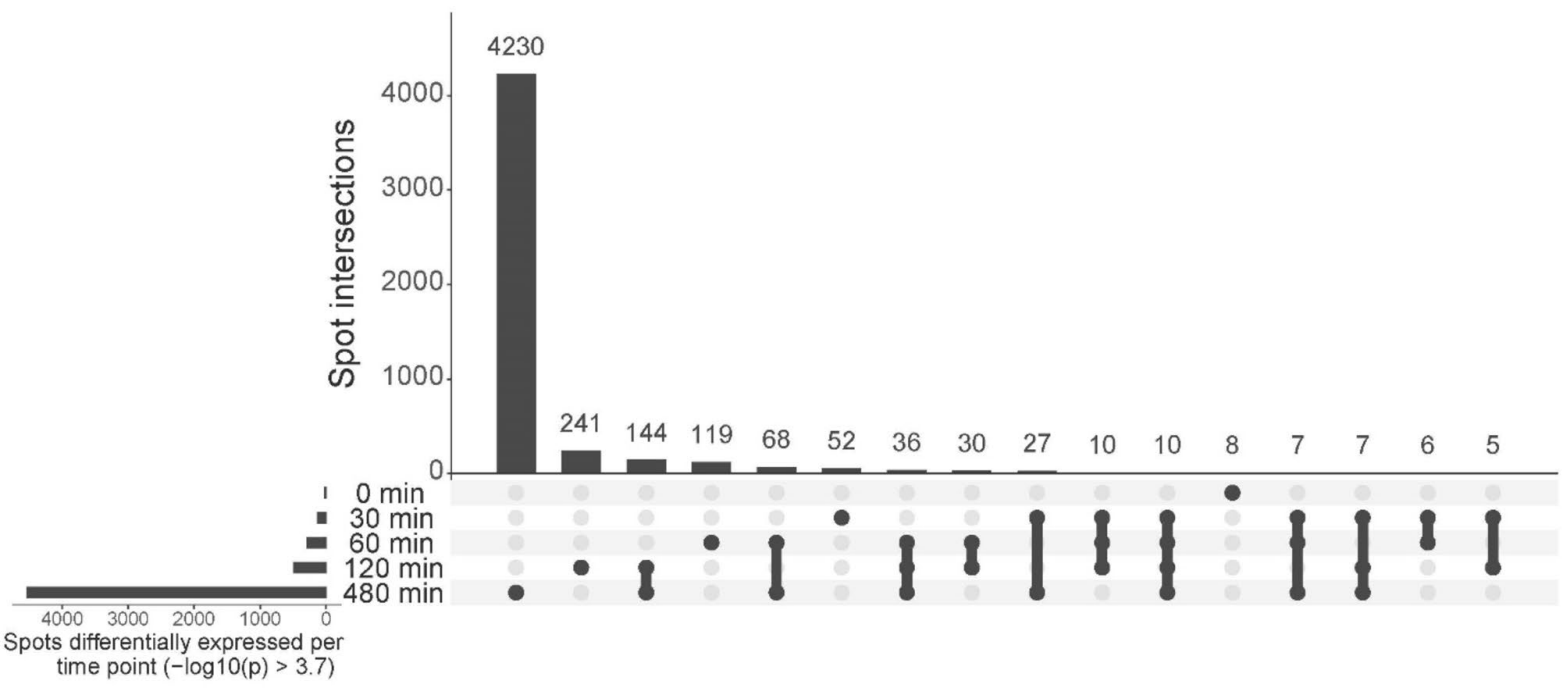
UpSet plot of the linear model. In the lower left corner, the total number of differentially expressed spots between 0 mM versus 400 mM ethanol exposed animals can be observed per time point (− log10(p) > 3.7). The dots, lines, and bars represent the overlap of differentially expressed spots between the various time-points. For example: at 480 min, 4230 spots are only differentially regulated between the two treatments; hence, 144 spots are differentially regulated between 0 and 400 mM ethanol at both 120 min and 480 min of exposure.

To associate gene expression with ethanol exposure over time, we used a linear model where we investigated the four replicates of each timepoint for gene expression differences between treatments (Supplemental Table 1). Differential gene expression increased over exposure time (Fig. 3, Supplemental Table 3). We noticed that the majority of genes were only differentially expressed at one time point, suggesting that those genes have a time specific function upon ethanol exposure. We also observed gene expression differences between treatments that are more stable over time as some genes continue to be differentially expressed at multiple time points (Fig. 3, Supplemental Table 4). We used the differentially expressed genes (DEGs) per timepoint in a gene ontology enrichment analysis (Supplemental Table 5).

**Figure 4:**
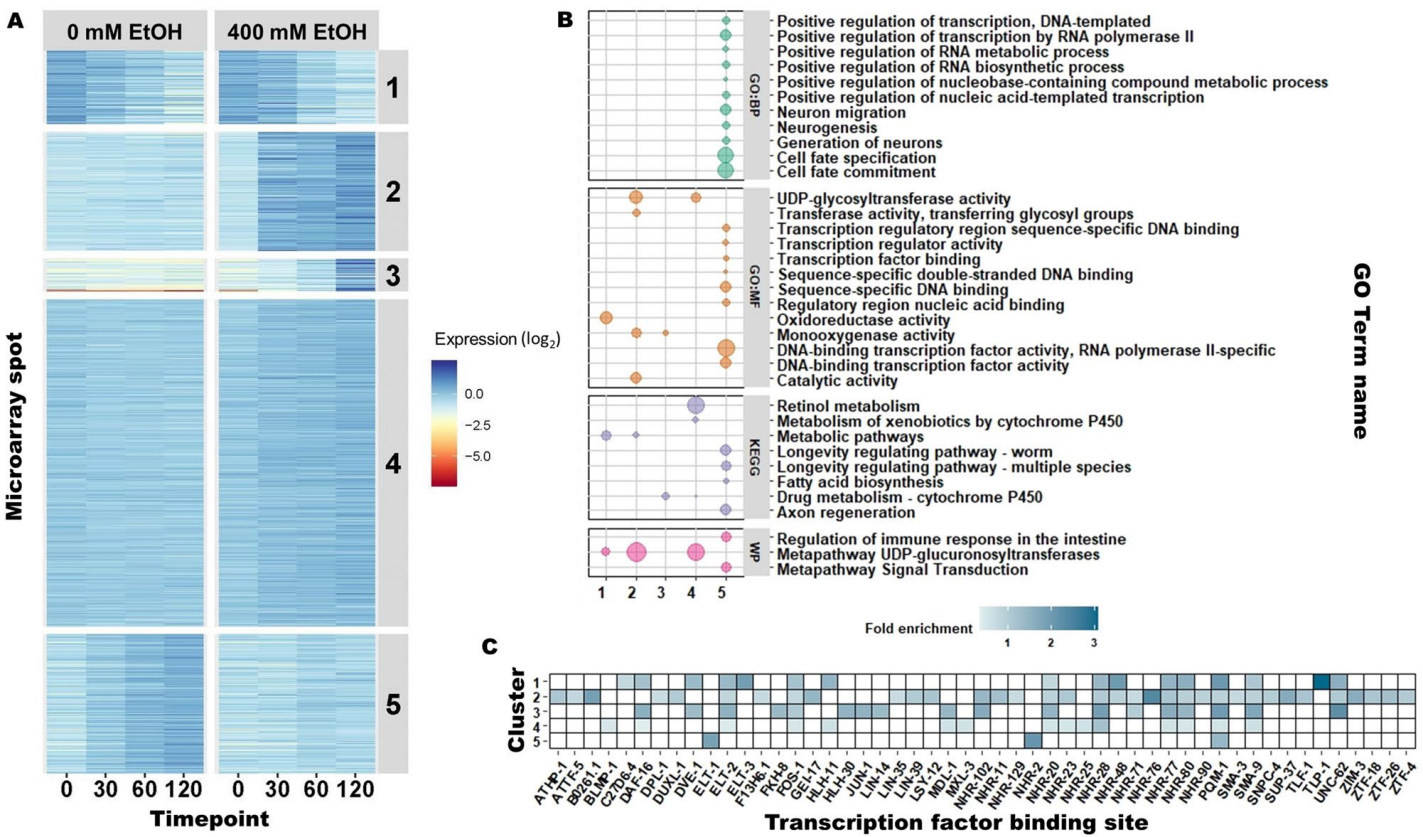
k-means clustering of differentially expressed genes. (**A**) differentially expressed genes clustered per expression pattern. Timepoints tested are on the x-axis and on the y-axis are the 959 microarray spots that represent differentially expressed genes (− log_10_(p) > 3.7). The differentially expressed genes were clustered in five groups by k-means clustering: (1) down-regulated over time, (2) early and strong up-regulation upon ethanol exposure, (3) late up-regulation upon ethanol exposure, (4) early and weak up-regulation upon ethanol exposure, and (5) unchanged in ethanol exposed animals, while increasing expression over time in the ethanol untreated animals (this can be interpreted as relatively down-regulated over time upon ethanol exposure). The colors represent the averaged expression over the four replicates per time and treatment combination (as log_2_ ratio with the mean). (**B**) Enrichment analysis of the subsequent k-clusters based on various annotations, including: GO-terms and KEGG pathways^88^. (**C**) Enrichment of transcription factor binding sites (TFBS) per K-means cluster. Fold enrichment is shown when the enrichment for the TFBS was significant (FDR < 0.05). The figure was created using R (version 3.5.3 × 64; https://cran.r-project.org/) and the ggplot2 package (version 3.3.3; https://ggplot2.tidyverse.org/).

We sought to understand the dynamics of ethanol-induced differential gene expression over time. For this analysis, we excluded the 480-min timepoint because of the major effect of the food deprivation at this timepoint (Fig. 2). For the determination of the appropriate number of clusters we used the “elbow” method^25^. We applied k-means clustering and clustered 959 spots (representing 660 significant differentially expressed genes) into five distinct patterns (Fig. 4A, Supplemental Table 6). The first cluster contained genes that were downregulated over time in both the 0 mM and 400 mM ethanol exposed animals; these were likely to be due to effects of the food deprivation. The second, third, and fourth clusters consist of genes upregulated in the 400 mM ethanol treated animals but unchanged in the 0 mM treated animals. The fifth cluster contains genes that were unchanged over time in the animals treated with 400 mM ethanol, but were upregulated over time in the 0 mM treated animals. We used these clusters in gene-enrichment analysis (Fig. 4B, Supplemental Table 7).

We asked if there were particular transcription factors that we could associate with ethanol-dependent gene regulation using a transcription factor binding site enrichment analysis. The cluster harboring genes that respond in the first 30 min to ethanol exposure was associated with the most transcription factor binding sites; these transcription factors include B0261.1, NHR-76, and SUP-37 (Fig. 4C). The enrichment of transcription factor binding sites is somewhat overlapping between the early response genes (Fig. 4A cluster 2) and genes that respond somewhat later (Fig. 4A cluster 3); among these are ELT-2, FOS-1, and NHR-102. However, we did find that there appears to be specificity to the ethanol-induced transcriptional regulation across time; we found that 40 of 51 associated transcription factor binding sites are different between those two clusters.

We reasoned that among the genes that are regulated in response to ethanol exposure would be genes that had already been identified as having roles in acute ethanol response phenotypes. We compiled a list of 77 genes that had been confirmed by behavioral testing in our and others’ laboratories to affect ethanol response behaviors in *C. elegans*^2,11,31,38–49^ and compared this list with the set of genes regulated at 480 min of ethanol exposure. We found that 21 of these genes were regulated at 480 min. (Supplemental Table 8). We focused primarily on genes whose expression was decreased in the ethanol-treated condition, with the idea that this should minimize the confounding effect of the food deprivation. Food deprivation appears in this study to decrease general transcription in the 0 mM treated animals but has less of an effect in 400 mM treated animals, which could lead to genes that are increased in the 400 mM exposure group incorrectly appearing to be regulated in response to ethanol.

## Discussion

In this study, we examined the transcriptional response of adult *C. elegans* to ethanol exposures of 30, 60, 120, and 480 min, with the goal of fully characterizing the acute effects of ethanol on gene expression. We treated age-synchronized young adult animals with 0 mM and 400 mM exogenous ethanol. This concentration of ethanol causes substantial behavioral effects; it results in significant loss of coordination and slowing of locomotion, as well as depression of egg laying^4–6,9^. We have previously found that over the course of a 60-min exposure to ethanol, only approximately 1/12th of the exogenous concentration of ethanol accumulates in the tissue of worms; this concentration is well within the range found in humans during heavy drinking episodes^50,51^.

To understand the biological implications of ethanol exposure we looked at time specific differential gene expression as well as gene expression patterns over time.

### Genes encoding serotonin signaling components are regulated in response to ethanol exposure

We found that different lengths of exposure to ethanol elicited distinct transcriptional profiles, and as expected, more genes were regulated at longer exposure times. Fewer than 100 genes were significantly regulated at 30 min and there were no overrepresented gene ontology categories, probably due to the small dataset. Interestingly, at this early timepoint, we observed an ethanol-induced increase in expression of *mod-1,* which encodes a serotonin-gated chloride channel (effect = − 0.19, FDR = 4.37 * 10^–2^). Serotonin signaling is a mechanism by which animals can modulate behavior based on external stimuli. In worms, serotonin activated MOD-1 channels play roles in the enhanced slowing response when worms that have been deprived of food encounter a new source of food^52–54^, and also in promoting a reduced exploration state called dwelling^55,56^; both are behavioral states that have reduced locomotion rates. It is interesting that a channel that is known to promote reduced locomotory states would be upregulated by exposure to ethanol at a concentration that depresses rates of locomotion; perhaps the pattern of *mod-1* expression is distinct, such that serotonin is inhibiting inhibitory neurons in the case of 30-min ethanol exposure. Serotonin is also implicated in alcohol use disorder in humans, although its role is complex and not completely understood. A genetic polymorphism in the serotonin reuptake transporter that increases serotonin in the synapse is associated with alcohol dependence, suggesting that increasing serotonin signaling promotes consumption^57^. This is consistent with the observation that people with alcohol use disorder have relatively greater expression of the serotonin biosynthetic enzyme tryptophan hydroxylase^58^. However, drugs that impact serotonin signaling have been shown to both increase and decrease ethanol drinking in different paradigms (reviewed in Marcinkiewcz et al*.* 2016^59^), leading to the suggestion that particular serotonin pathways are associated with different aspects of alcohol use phenotypes. It is notable that we observe that among the, at least, four serotonin receptors in worms, only *mod-1* was regulated at this timepoint, indicating that there is specificity in the ethanol responsiveness of this neurotransmitter pathway. We note that at the 480 min timepoint, there was downregulation of both the *bas-1* (dopa decarboxylase) (effect = − 0.43, FDR = 2.06 * 10^–4^) and *cat-1* (catecholamine symporter) (effect = − 0.19, FDR = 2.12 * 10^–3^) enzymes that are involved in regulating serotonin levels, suggesting that by this point, a global decrease in serotonin signaling is induced by ethanol.

### Metabolic enzymes and transcriptional regulators are regulated during middle time points of ethanol exposure

At 60 min of exposure, 220 genes were significantly regulated. As expected, overrepresented in this group are genes involved in alcohol metabolism (alcohol dehydrogenase activity). In addition, there was robust regulation of transcription factors; these are excellent candidate mediators of subsequent downstream expression changes, and suggest that by this time of exposure, a longer-term response to the effects of ethanol is evident. At 120 min of ethanol exposure, 379 genes were significantly regulated, and cytochrome P450 family members are overrepresented in the gene set. Cytochrome P450s are metabolic enzymes and are induced in many systems in response to drug treatments, including alcohol^60^.

### Eight hours of ethanol exposure causes regulation of genes involved in neuronal function and genes encoding direct molecular targets of ethanol

At eight hours of ethanol exposure, we observed the largest number of and most diverse changes in gene expression; we found significant changes in expression of 2938 genes. There is a large effect of the experimental treatment at this timepoint independent of the ethanol concentration. We interpret this observation as the transcriptional response to food deprivation, which is considerably, although not completely, ameliorated by the presence of ethanol. This somewhat confounds the interpretation of the biological significance of the differentially expressed genes that are due to ethanol at this timepoint, but because our overall goal with this work is to identify the long-lasting adaptive changes that occur upon exposure to ethanol, we examined the biological actions of DEGs at this timepoint. We paid particular attention to genes whose expression was down in the ethanol-treated animals relative to the non-ethanol treated animals, because these may be less likely to reflect the potential moderating effect of ethanol on starvation-induced global down regulation of transcription.

We found that among the overrepresented classes of genes were those involved in locomotory behavior and synaptic transmission. For example, the genes encoding the synaptic active zone protein, UNC-13 (effect = − 0.56, FDR = 1.41 * 10^–3^), and its binding partner, UNC-10 (effect = − 0.68, FDR = 5.42 * 10^–3^) were both downregulated in ethanol treated animals. A mouse homolog of UNC-13, Munc13-1, has been demonstrated to be a direct molecular target of ethanol^61^. In *Drosophila*, reducing the levels of the *unc-13* homolog Dunc13 generates resistance to the depressing effects of ethanol on synaptic transmission^62^.

Genes encoding several components of the GABA signaling pathway are also regulated, including *unc-25* (glutamate decarboxylase) (effect = − 0.59, FDR = 1.41 * 10^–4^), *unc-47* (GABA transporter) (effect = − 0.72, FDR = 2.04 * 10^–4^), and *unc-49* (GABA receptor) (effect = − 0.54, FDR = 9.61 * 10^–4^), all of which were down-regulated with 8 h of ethanol exposure. The mammalian GABA_A_ receptor is a well-described direct target of ethanol, and ethanol’s action as a positive allosteric modulator of GABA_A_ receptors is an important component of the intoxicating effects of ethanol. The GABA system plays important roles in both acute and chronic consumption of ethanol^63^. Our observation that the GABA system is regulated with exposure to ethanol is consistent with an extensive literature in mammalian brains revealing that there is a complex manipulation of expression of GABA signaling components with ethanol treatments (reviewed in Roberto and Varodayan, 2017)^64^. This suggests that, like mammals, worms use transcriptional regulation of GABA system components to respond to ethanol exposure.

We and others have previously demonstrated that the BK potassium channel SLO-1 is a direct target of ethanol in worms and mammals^4,65,66^ and, in worms, is a major contributor to the depressive effects of ethanol^4^. We found that expression of the BK encoding gene *slo-1* was decreased with 8 h of ethanol exposure (effect = 1.11, FDR = 5.79 * 10^–4^). These data are consistent with the observation in rats that ethanol causes a rapid downregulation of BK channel mRNA in supraoptic nucleus neurons^67^, and suggests a simple model in which downregulation of *slo-1* and other direct ethanol targets could decrease the depressing effects of ethanol and, as such, could contribute to the development of tolerance to ethanol.

### Eight hours of ethanol exposure causes regulation of genes known to be involved in ethanol response behaviors

We hypothesized that in addition to *slo-1*, some of the genes that are regulated in response to ethanol exposure would already have been identified as having roles in acute ethanol response phenotypes. We have previously demonstrated that regulation of lipid levels is profoundly important in the process of development of acute functional tolerance to ethanol (AFT)^6,7,68^. We found that the triacylglyceride lipase gene, *lips-7* was downregulated at 8 h of ethanol exposure (effect = − 0.56, FDR = 9.06 * 10^–4^). *lips-7* acts antagonistically to the development of AFT; *lips-7* loss of function mutants have enhanced development of AFT^6^. This suggests that down-regulation of *lips-7* transcription after extended exposure to ethanol may enhance the development of tolerance to ethanol.

In addition to triacylglycerides, the level of ω-3 polyunsaturated fatty acid eicosapentaenoic acid (EPA) also regulates the development of AFT. Depletion of EPA eliminates AFT, whereas supplementing additional dietary EPA enhances normal AFT in worms^7^, and in mice, altering EPA levels in diet modifies both acute ethanol responses and voluntary consumption^68^. The *fat-1* gene encodes a desaturase that generates EPA from arachidonic acid, and we found that the expression of *fat-1* was significantly increased after 8 h of ethanol exposure (effect = 0.25, FDR = 1.39 * 10^–3^). If this upregulation is a compensatory response to the effects of ethanol, then this suggests that increasing the amount of EPA may be an additional mechanism by which animals modify their systems to generate tolerance to extended ethanol exposure.

### Eight hours of ethanol exposure causes regulation of genes involved in cilia function

We were intrigued to observe that at 8 h of exposure, one of the most over-represented classes of DEGs included genes involved in cilia maintenance and function. In humans, there are significant effects of chronic ethanol exposure on cilia, and these effects underlie, at least in part, the profound dysfunction in lungs observed in many people who chronically abuse alcohol. Respiratory illness is an important cause of alcohol-related mortality; people with AUD are significantly more susceptible to serious pulmonary infections, as well as at higher risk of acute respiratory distress syndrome (ARDS), an often-fatal fluid buildup in the alveoli (reviewed in Simet and Sisson, 2015)^69^. Alcohol Induced Ciliary Dysfunction (AICD) is one of the root causes of lung dysfunction because it disrupts the ability of the ciliated airways to move inhaled particles and pathogens out of the lungs. AICD is associated with the disruption of the regulation of cilia beating frequency, and this effect has been linked to the outer dynein arm of lung cilia^70,71^. Notably, we found that the *C. elegans* ortholog of the human dynein heavy chain, *che-3,* was significantly downregulated after 8 h of ethanol exposure (effect = − 0.96, FDR = 1.10 * 10^–4^).

Recent work suggests that cilia dysfunction caused by alcohol may also underlie some of alcohol’s effects on the nervous system. Excess alcohol consumption is associated with a buildup of cerebral spinal fluid (CSF) resulting in hydrocephalus, which can cause pressure related brain damage^72^. Fetal alcohol exposure can cause hydrocephalus^73–75^, which can result in lasting consequences to the affected child. Al Omran et al*.* (2017) recently demonstrated that acute alcohol exposure in rat causes a decrease in the beating frequency of the ependymal cilia lining in the brain that are involved in moving CSF^76^. Our work raises the intriguing possibility that the regulation of cilia gene expression may be a response to the inhibition of cilia function that occurs during extended ethanol exposure.

This study examined the effects of a time course that culminated in 8 h of exposure to 400 mM ethanol. There have been at least two other examinations of the transcriptomics effects of exposure to ethanol in *C. elegans* using quite different exposure conditions. Kwon et al*.* (2004)^77^ performed a microarray analysis at 15 min, 30 min, and 6 h of exposure to a high 1.5 M concentration of ethanol (they found that this concentration is lethal after 6 h of exposure). Moreover, this study has only 2 or 1 replicates at 15 min and 30 min respectively, which may reduce the ability to eliminate false positives or detect more subtle expression changes. This study reported that the only biological category of genes whose expression increased in ethanol treatment was the heat shock protein family. Interestingly, in our study, several of these heat shock protein genes were not upregulated (in fact, we found that several were downregulated), suggesting that this heat shock response is characteristic of the very high concentration of ethanol used in the Kwon et al. study. Of the 230 genes identified in this study as being ethanol responsive, we found that 21 were similarly regulated in our study, suggesting that these are responding to the ethanol exposure regardless of the concentration of ethanol experienced but consistent with exposure time. Peltonen et al. (2013)^78^ performed an RNAseq analysis of a chronic developmental exposure to a lower ethanol concentration (200 mM). In this work, two analyses were performed; developing larvae were exposed to ethanol for 2 days throughout postembryonic development and were harvested before adulthood, and a second group were exposed for 7 days and harvested at approximately 4 days of adulthood. This group observed that approximately 1000 genes were regulated in response to ethanol over these long developmental times. Among the most overrepresented gene classes were those involved in cytochrome P450 drug metabolism. Our observation that the P450 enzymes were regulated in our studies suggests that this is a general ethanol response mechanism.

### k-means clustering

An additional approach to understanding gene expression dynamics is to consider genes that are affected by the treatment in the same pattern across the samples because co-expression of genes can shed light on specific biological processes that are affected by treatment. We excluded the 8-h timepoint from this analysis to minimize the effect of starvation. We performed k-means clustering of the 959 spots that were significantly different in the 0-, 30-, 60-, and 120-min exposures and grouped them into 5 clusters, each of which had a distinct pattern over time. We performed an enrichment analysis for each cluster to see if there were processes and pathways that were overrepresented that might highlight important functions underlying the ethanol response over time^79–81^.

Cluster 1 contained genes that were down-regulated over time regardless of treatment; these are likely to reflect the transcriptional response to the food deprivation during the exposure, and are unlikely to be informative about the ethanol response. Cluster 2 consisted of genes that were upregulated in the early timepoints and were progressively more upregulated with longer exposures to ethanol. This cluster was enriched for pathways that are involved in glycosylation. Glycosylation pathways are important for posttranslational modifications^82^. Interestingly, alcohol consumption affects glycosylation levels^83^, and moderate alcohol consumption is associated with exacerbation of antithrombin deficiency in individuals with disorders of glycosylation^84^. In one study, in children diagnosed with fetal alcohol syndrome there were more incidents of alleles compromising function of N-glycosylation machinery, suggesting that decreasing function of N-glycosylation may increase prenatal sensitivity to the teratogenic effects of ethanol^85^.

Cluster 3 contained genes that were strongly upregulated at later timepoints during the ethanol exposure; this small cluster contained enzymes involved in drug metabolism. Genes in cluster 4 were weakly upregulated across the ethanol exposure timecourse; genes in this cluster are involved in multiple processes including retinol (vitamin A) metabolism. There is a complicated relationship between retinols and ethanol; enzymes that metabolize ethanol appear to have an impact on retinol metabolism and homeostasis, and ethanol drinking dysregulates retinol metabolism; this has important negative health consequences in people with AUD (reviewed by Clugston and Blaner, 2012)^86^. In addition, alcohol induced dysregulation of retinol metabolism may contribute to the development of fetal alcohol syndrome^87^.

Cluster 5 consisted of genes whose expression was upregulated over time in animals not exposed to ethanol, presumably in response to the food deprivation during the assay. However, in animals exposed to ethanol, these genes were stably expressed, suggesting that ethanol suppressed the starvation-induced expression change. Among the over-represented classes were genes involved in neuronal function; one intriguing possibility is that these genes contribute to neuroadaptations to the food deprivation stress that are inhibited by ethanol.

In conclusion, we have identified ethanol exposure-induced transcriptional regulation of genes involved in pathways predicted by our previous work, including several direct targets of ethanol and genes involved in alcohol and lipid metabolism, as well as novel gene groups, including those involved in cilia function, glycosylation and retinol metabolism. Regulation of these genes in humans by ethanol exposure may have significant consequences for the long-term abuse of the drug and health-related consequences of alcohol consumption.

## Supplementary Information

Supplementary Information.

Supplementary Legend.

Supplementary Figure 1.

## References

[1] *Global Status Report on Alcohol and Health 2018. Licence: CC BY-NC-SA 3.0 IGO*. World Health Organization (2018).

[2] Grotewiel M, Bettinger JC (2015). *Drosophila* and *Caenorhabditis elegans* as discovery platforms for genes involved in human alcohol use disorder. Alcohol. Clin. Exp. Res..

[3] Alaimo JT (2012). Ethanol metabolism and osmolarity modify behavioral responses to ethanol in *C. elegans*. Alcohol. Clin. Exp. Res..

[4] Davies AG (2003). A central role of the BK potassium channel in behavioral responses to ethanol in *C. elegans*. Cell.

[5] Davies AG, Bettinger JC, Thiele TR, Judy ME, McIntire SL (2004). Natural variation in the *npr-1* gene modifies ethanol responses of wild strains of *C. elegans*. Neuron.

[6] Bettinger JC, Leung K, Bolling MH, Goldsmith AD, Davies AG (2012). Lipid environment modulates the development of acute tolerance to ethanol in *Caenorhabditis elegans*. PLoS ONE.

[7] Raabe RC, Mathies LD, Davies AG, Bettinger JC (2014). The omega-3 fatty acid eicosapentaenoic acid is required for normal alcohol response behaviors in *C. elegans*. PLoS ONE.

[8] Hawkins EG (2015). A novel cholinergic action of alcohol and the development of tolerance to that effect in *Caenorhabditis elegans*. Genetics.

[9] Mathies LD (2015). SWI/SNF chromatin remodeling regulates alcohol response behaviors in *Caenorhabditis elegans* and is associated with alcohol dependence in humans. Proc. Natl. Acad. Sci. USA.

[10] Mitchell P (2010). A differential role for neuropeptides in acute and chronic adaptive responses to alcohol: Behavioural and genetic analysis in *Caenorhabditis elegans*. PLoS ONE.

[11] Scott LL (2017). Behavioral deficits following withdrawal from chronic ethanol are influenced by SLO channel function in *Caenorhabditis elegans*. Genetics.

[12] Brenner S (1974). The genetics of *Caenorhabditis elegans*. Genetics.

[13] Jovic K (2017). Temporal dynamics of gene expression in heat-stressed *Caenorhabditis elegans*. PLoS ONE.

[14] Ritchie ME (2015). Limma powers differential expression analyses for RNA-sequencing and microarray studies. Nucleic Acids Res.

[15] Zahurak M (2007). Pre-processing Agilent microarray data. BMC Bioinform..

[16] Smyth GK, Speed T (2003). Normalization of cDNA microarray data. Methods.

[17] R Core Team 3.6.2. *R: A Language and Environment for Statistical Computing*. R Foundation for Statistical Computing. Vienna, Austria. http://www.R-project.org/ (2019).

[18] Wickham, H. & Henry, L. *tidyr: Easily Tidy Data with ‘spread()’ and ‘gather()’ Functions. R Packag. Version 0.8.0*. https://CRAN.R-project.org/package=tidyr (2018).

[19] Wickham, H., François, R., Henry, L. & Müller, K. *dplyr: A Grammar of Data Manipulation. R Package Version* (2019).

[20] Wickham H (2009). ggplot2: Elegant Graphics for Data Analysis.

[21] Snoek BL (2020). WormQTL2: An interactive platform for systems genetics in *Caenorhabditis elegans*. Database (Oxford).

[22] Sterken MG (2014). A heritable antiviral RNAi response limits orsay virus infection in *Caenorhabditis elegans* N2. PLoS ONE.

[23] Benjamini Y, Hochberg Y (1995). Controlling the false discovery rate: A practical and powerful approach to multiple testing. J. R. Stat. Soc. Ser. B.

[24] Snoek LB (2014). A rapid and massive gene expression shift marking adolescent transition in *C. elegans*. Sci. Rep..

[25] Kassambara A (2017). Practical Guide to Cluster Analysis in R—Unsupervised Machine Learning.

[26] Charrad M, Ghazzali N, Boiteau V, Niknafs A (2014). NbClust: An R package for determining the relevant number of clusters in a data set. J. Stat. Softw..

[27] Lee RYN (2018). WormBase 2017: Molting into a new stage. Nucleic Acids Res..

[28] Kudron MM (2018). The modern resource: genome-wide binding profiles for hundreds of *Drosophila* and *Caenorhabditis elegans* transcription factors. Genetics.

[29] Tepper RG (2013). PQM-1 complements DAF-16 as a key transcriptional regulator of DAF-2-mediated development and longevity. Cell.

[30] Raudvere U (2019). G:Profiler: A web server for functional enrichment analysis and conversions of gene lists (2019 update). Nucleic Acids Res..

[31] Adkins AE (2017). Genomewide association study of alcohol dependence identifies risk loci altering ethanol-response behaviors in model organisms. Alcohol Clin Exp Res.

[32] Harvald EB (2017). Multi-omics analyses of starvation responses reveal a central role for lipoprotein metabolism in acute starvation survival in *C. elegans*. Cell Syst..

[33] Davis JR, Li Y, Rankin CH (2008). Effects of developmental exposure to ethanol on *Caenorhabditis elegans*. Alcohol. Clin. Exp. Res..

[34] Cooper AF, Van Gundy SD (1971). Ethanol production and utilization by *Aphelenchus avenae* and *Caenorhabditis *sp. J. Nematol..

[35] Castro PV, Khare S, Young BD, Clarke SG (2012). *Caenorhabditis elegans* battling starvation stress: Low levels of ethanol prolong lifespan in L1 Larvae. PLoS ONE.

[36] Patananan AN, Budenholzer LM, Eskin A, Torres ER, Clarke SG (2015). Ethanol-induced differential gene expression and acetyl-CoA metabolism in a longevity model of the nematode *Caenorhabditis elegans*. Exp. Gerontol..

[37] Kaptan D (2020). Exogenous ethanol induces a metabolic switch that prolongs the survival of *Caenorhabditis elegans* dauer larva and enhances its resistance to desiccation. Aging Cell.

[38] Johnson JR, Rajamanoharan D, McCue H, Rankin K, Barclay JW (2016). Small heat shock proteins are novel common determinants of alcohol and nicotine sensitivity in *Caenorhabditis elegans*. Genetics.

[39] Oh KH (2017). ERG-28 controls BK channel trafficking in the ER to regulate synaptic function and alcohol response in *C. elegans*. Elife.

[40] Johnson JR (2017). Ethanol stimulates locomotion via a g_αs_-signaling pathway in IL2 neurons in *Caenorhabditis elegans*. Genetics.

[41] Chen YH (2018). GCY-35/GCY-36 - TAX-2/TAX-4 signalling in O_2_ sensory neurons mediates acute functional ethanol tolerance in *Caenorhabditis elegans*. Sci. Rep..

[42] Scott LL (2018). Small molecule modulators of σ2R/Tmem97 reduce alcohol withdrawal-induced behaviors. Neuropsychopharmacology.

[43] Katner SN (2019). *Caenorhabditis elegans* as a model system to identify therapeutics for alcohol use disorders. Behav. Brain Res..

[44] Oh KH, Kim H (2019). BK channel clustering is required for normal behavioral alcohol sensitivity in *C. elegans*. Sci. Rep..

[45] Maulik M (2019). Genetic silencing of fatty acid desaturases modulates α-synuclein toxicity and neuronal loss in Parkinson-like models of *C. elegans*. Front. Aging Neurosci..

[46] Thompson A (2020). Functional validity, role, and implications of heavy alcohol consumption genetic loci. Sci. Adv..

[47] Oh KH, Sheoran S, Richmond JE, Kim H (2020). Alcohol induces mitochondrial fragmentation and stress responses to maintain normal muscle function in *Caenorhabditis elegans*. FASEB J..

[48] Mathies LD (2020). SWI/SNF complexes act through CBP-1 histone acetyltransferase to regulate acute functional tolerance to alcohol. BMC Genomics.

[49] Pandey P, Singh A, Kaur H, Ghosh-Roy A, Babu K (2021). Increased dopaminergic neurotransmission results in ethanol dependent sedative behaviors in *Caenorhabditis elegans*. PLoS Genet..

[50] Bond J (2010). The relationship between self-reported drinking and BAC level in emergency room injury cases: Is it a straight line?. Alcohol. Clin. Exp. Res..

[51] Vonghia L (2008). Acute alcohol intoxication. Eur. J. Intern. Med..

[52] Sawin ER, Ranganathan R, Horvitz HRC (2000). *elegans* locomotory rate is modulated by the environment through a dopaminergic pathway and by experience through a serotonergic pathway. Neuron.

[53] Ranganathan R, Cannon SC, Horvitz HR (2000). MOD-1 is a serotonin-gated chloride channel that modulates locomotory behaviour in *C. elegans*. Nature.

[54] Gürel G, Gustafson MA, Pepper JS, Robert Horvitz H, Koelle MR (2012). Receptors and other signaling proteins required for serotonin control of locomotion in *Caenorhabditis elegans*. Genetics.

[55] Flavell SW (2013). Serotonin and the neuropeptide PDF initiate and extend opposing behavioral states in *C. elegans*. Cell.

[56] Fujiwara M, Sengupta P, Mcintire SL (2002). Regulation of body size and behavioral state of *C. elegans* by sensory perception and the EGL-4 cGMP-dependent protein kinase. Neuron.

[57] Feinn R, Nellissery M, Kranzler HR (2005). Meta-analysis of the association of a functional serotonin transporter promoter polymorphism with alcohol dependence. Am. J. Med. Genet. Neuropsychiatr. Genet..

[58] Bonkale WL, Turecki G, Austin MC (2006). Increased tryptophan hydroxylase immunoreactivity in the dorsal raphe nucleus of alcohol-dependent, depressed suicide subjects is restricted to the dorsal subnucleus. Synapse.

[59] Marcinkiewcz CA, Lowery-Gionta EG, Kash TL (2016). Serotonin’s complex role in alcoholism: Implications for treatment and future research. Alcohol. Clin. Exp. Res..

[60] Peter-Guengerich F, Avadhani NG, Vasiliou V, Zakhari S, Mishra L, Seitz H (2018). Roles of cytochrome P450 in metabolism of ethanol and carcinogens. Alcohol and Cancer. Advances in Experimental Medicine and Biology.

[61] Das J (2013). The pre-synaptic Munc13-1 binds alcohol and modulates alcohol self-administration in *Drosophila*. J. Neurochem..

[62] Xu S (2018). Ethanol regulates presynaptic activity and sedation through presynaptic Unc13 proteins in Drosophila. eNeuro.

[63] Koob GF (2009). Neurobiological substrates for the dark side of compulsivity in addiction. Neuropharmacology.

[64] Roberto M, Varodayan FP (2017). Synaptic targets: Chronic alcohol actions. Neuropharmacology.

[65] Davis SJ, Scott LL, Hu K, Pierce-Shimomura JT (2014). Conserved single residue in the BK potassium channel required for activation by alcohol and intoxication in *C. elegans*. J. Neurosci..

[66] Bukiya AN (2014). An alcohol-sensing site in the calcium- and voltage-gated, large conductance potassium (BK) channel. Proc. Natl. Acad. Sci. USA.

[67] Pietrzykowski AZ (2008). Posttranscriptional regulation of BK channel splice variant stability by miR-9 underlies neuroadaptation to alcohol. Neuron.

[68] Wolstenholme JT (2018). Dietary omega-3 fatty acids differentially impact acute ethanol-responsive behaviors and ethanol consumption in DBA/2J versus C57BL/6J mice. Alcohol. Clin. Exp. Res..

[69] Simet SM, Sisson JH (2015). Alcohol’s effects on lung health and immunity. Alcohol. Res..

[70] Yang F (2015). Alcohol-induced ciliary dysfunction targets the outer dynein arm. Am. J. Physiol. Lung Cell. Mol. Physiol..

[71] Price M, Yang F, Sisson JH, Wirschell M, King SM (2018). Ciliary dynein dysfunction caused by chronic alcohol exposure. Dyneins: Structure, Biology and Disease: Dynein Mechanics, Dysfunction, and Disease.

[72] Hickman TT (2017). Association between shunt-responsive idiopathic normal pressure hydrocephalus and alcohol. J. Neurosurg..

[73] De La Monte SM, Kril JJ (2014). Human alcohol-related neuropathology. Acta Neuropathol..

[74] Sakata-Haga H, Sawada K, Ohnishi T, Fukui Y (2004). Hydrocephalus following prenatal exposure to ethanol. Acta Neuropathol..

[75] Jarmasz JS, Basalah DA, Chudley AE, Del Bigio MR (2017). Human brain abnormalities associated with prenatal alcohol exposure and fetal alcohol spectrum disorder. J. Neuropathol. Exp. Neurol..

[76] Omran AJA (2017). Alcohol consumption impairs the ependymal cilia motility in the brain ventricles. Sci. Rep..

[77] Kwon JY (2004). Ethanol-response genes and their regulation analyzed by a microarray and comparative genomic approach in the nematode *Caenorhabditis elegans*. Genomics.

[78] Peltonen J, Aarnio V, Heikkinen L, Lakso M, Wong G (2013). Chronic ethanol exposure increases cytochrome P-450 and decreases activated in blocked unfolded protein response gene family transcripts in *Caenorhabditis elegans*. J. Biochem. Mol. Toxicol..

[79] Nilsson R (2009). Discovery of genes essential for heme biosynthesis through large-scale gene expression analysis. Cell Metab..

[80] Pierson E, Koller D, Battle A, Mostafavi S (2015). Sharing and specificity of co-expression networks across 35 human tissues. PLoS Comput. Biol..

[81] Jiménez-Ruiz J, de la O Leyva-Pérez M, Vidoy-Mercado I, Barceló A, Luque F (2018). Transcriptomic time-series analysis of early development in olive from germinated embryos to juvenile tree. BMC Genomics.

[82] Zielinska DF, Gnad F, Schropp K, Wiśniewski JR, Mann M (2012). Mapping N-glycosylation sites across seven evolutionarily distant species reveals a divergent substrate proteome despite a common core machinery. Mol. Cell.

[83] Waszkiewicz N (2012). Alcohol abuse and glycoconjugate metabolism. Folia Histochem. Cytobiol..

[84] de la Morena-Barrio ME (2016). Hypoglycosylation is a common finding in antithrombin deficiency in the absence of a SERPINC1 gene defect. J. Thromb. Haemost..

[85] De La Morena-Barrio ME (2018). Genetic predisposition to fetal alcohol syndrome: Association with congenital disorders of N-glycosylation. Pediatr. Res..

[86] Clugston RD, Blaner WS (2012). The adverse effects of alcohol on vitamin A metabolism. Nutrients.

[87] Petrelli B, Bendelac L, Hicks GG, Fainsod A (2019). Insights into retinoic acid deficiency and the induction of craniofacial malformations and microcephaly in fetal alcohol spectrum disorder. Genesis.

[88] Kanehisa M, Furumichi M, Sato Y, Ishiguro-Watanabe M, Tanabe M (2021). KEGG: Integrating viruses and cellular organisms. Nucleic Acids Res..

