# Supplementary material for: Transcriptional analysis of the response of C. elegans to ethanol exposure
Source: Sci Rep. 2021 May 26;11:10993. doi: 10.1038/s41598-021-90282-8 (PMC8155136; doi:10.1038/s41598-021-90282-8)
Supplement: Supplementary file 2 — Supplementary Legend. [file 41598_2021_90282_MOESM2_ESM.docx]

**Supplemental Figure 1: qRT-PCR of selected genes in response to ethanol.** qPCR was performed on the same samples that were used for microarray analysis. The results of the microarray and qPCR show the same expression changes for the selected genes, although in this analysis, we did not find statistically significant expression differences for mod-1 or unc-49. Significance was determined by ANOVA; * p ≤ 0.05, ** p ≤ 0.01, *** p ≤ 0.001, **** p ≤ 0.0001. qPCR analysis was performed with n=4, except for unc-49 exposed to 0 mM and unc-47 exposed to 400 mM that are n=3. **A.** Expression pattern of genes from samples that were exposed to 0 mM or 400 mM for 30 minutes. **B.** Expression pattern of genes from samples that were exposed to 0 mM or 400 mM ethanol for 480 minutes.
