# Supplementary figures and images for: Transcriptional analysis of the response of C. elegans to ethanol exposure
Source: Sci Rep. 2021 May 26;11:10993. doi: 10.1038/s41598-021-90282-8 (PMC8155136; doi:10.1038/s41598-021-90282-8)

Ethanol concentration (mM) 0 400

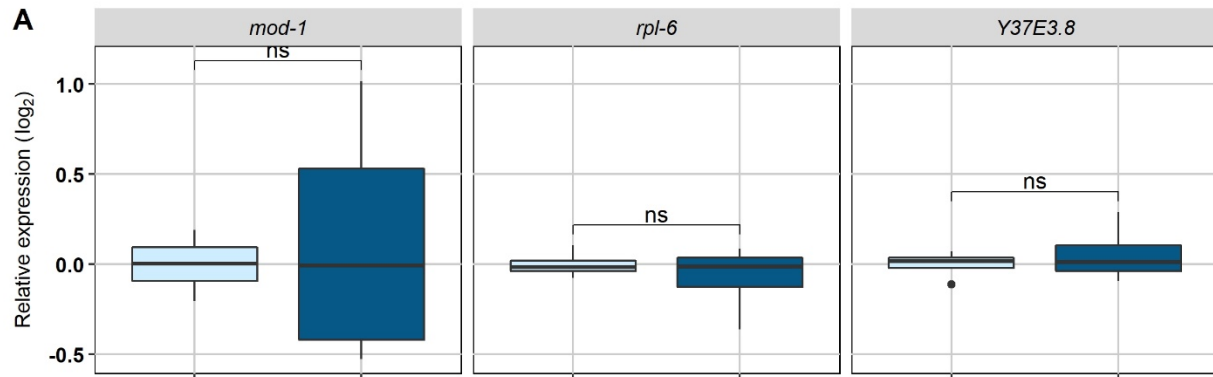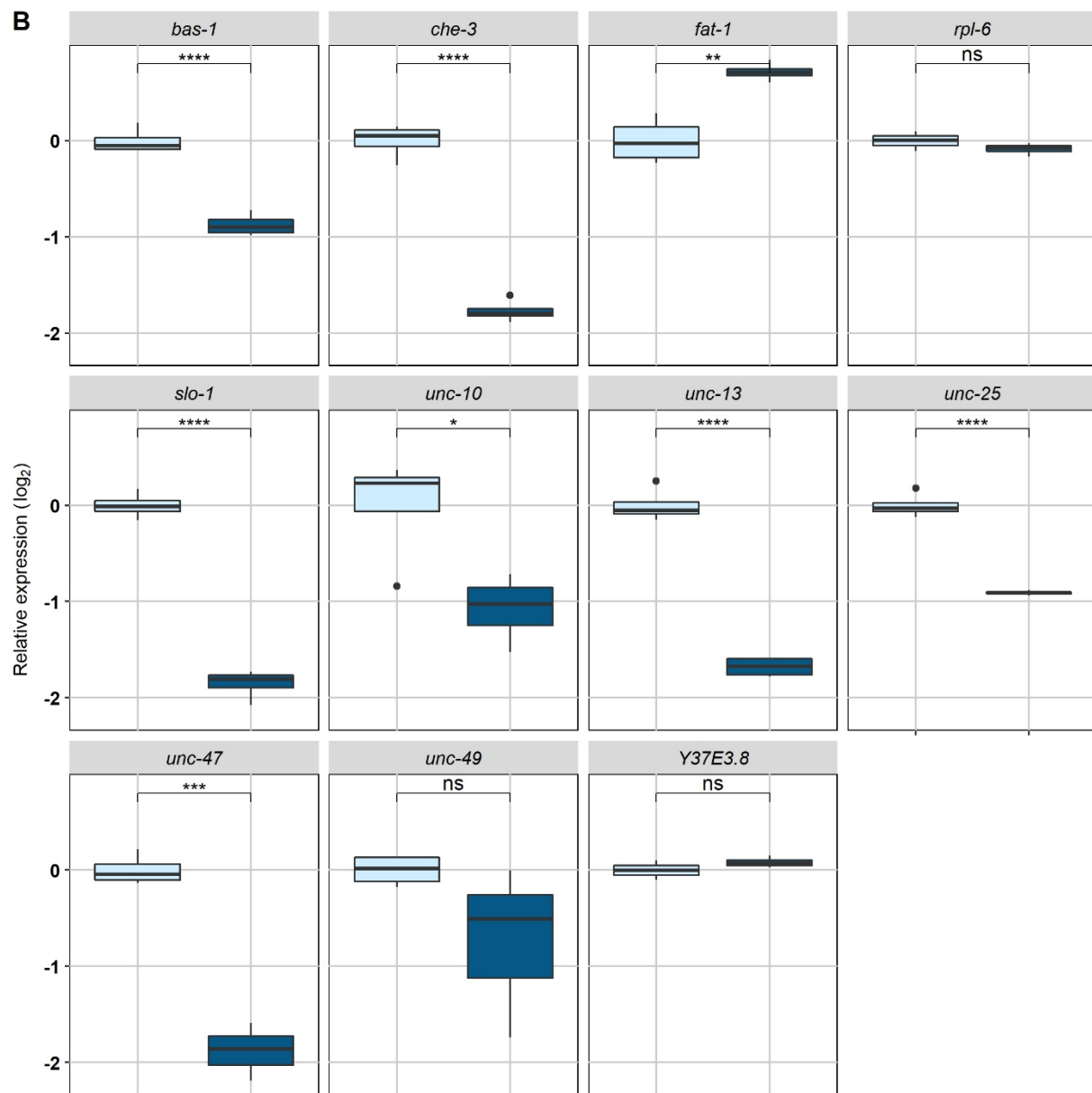

Supplement: Supplementary file 3 — Supplementary Figure 1. [file 41598_2021_90282_MOESM3_ESM.pdf]
